# Supplementary material for: Emerging Pharmacological Interventions for Chronic Venous Insufficiency: A Comprehensive Systematic Review and Meta-Analysis of Efficacy, Safety, and Therapeutic Advances
Source: Pharmaceutics. 2025 Jan 3;17(1):59. doi: 10.3390/pharmaceutics17010059 (PMC11768779; doi:10.3390/pharmaceutics17010059)
Supplement: Supplementary file 1 [file pharmaceutics-17-00059-s001.zip › pharmaceutics-3380701-supplementary.pdf]

pharmaceutics

MDPI

**Supplementary Materials:**

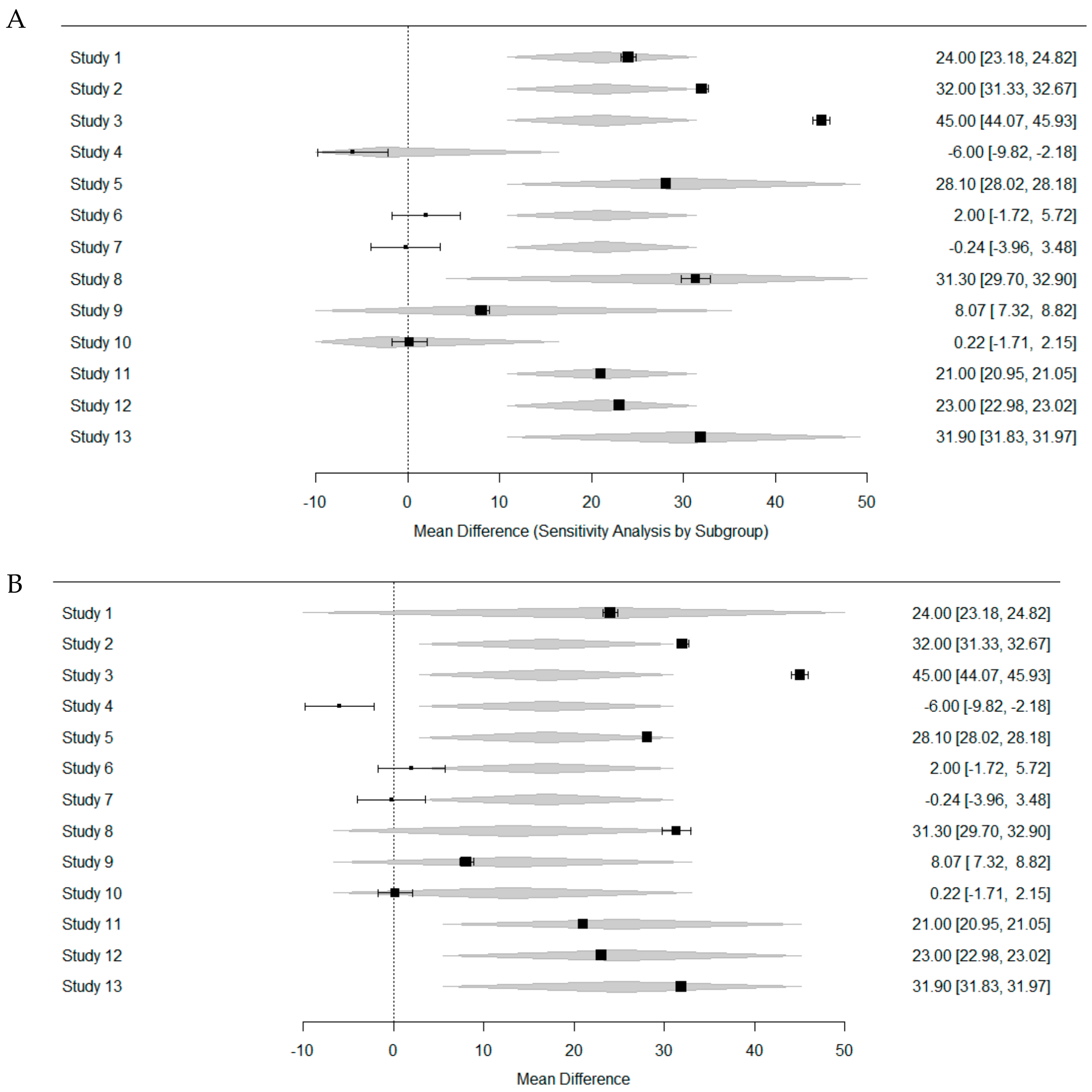


**Figure S1**. Sensitivity analyses for chronic venous insufficiency interventions. (a) Mixed-effects meta-analysis stratified by intervention types (Coumarin + Troxerutin, Hydroxyethylrutoside, Pycnogenol, and Venoruton). The x-axis shows the mean difference (MD) with 95% confidence intervals, and the y-axis lists data points corresponding to various interventions. The analysis reveals persistent residual heterogeneity ($\tau^2 = 190.70$; $I^2 = 100\%$), with no statistically significant differences among subgroups (QM = 7.78, p = 0.0999). (b) Mixed-effects meta-analysis stratified by clinical manifestations (Pain, Quality of Life, and Resting Flux). The x-axis shows the MD with 95% confidence intervals, and the y-axis lists data points corresponding to different manifestations. The analysis indicates no significant moderating effect of clinical manifestations on heterogeneity ($R^2 = 0.00\%$; QM = 0.88, p = 0.8313), with residual heterogeneity remaining high ($\tau^2 = 306.11$; $I^2 = 100\%$).

 https://doi.org/10.3390/pharmaceutics17010059

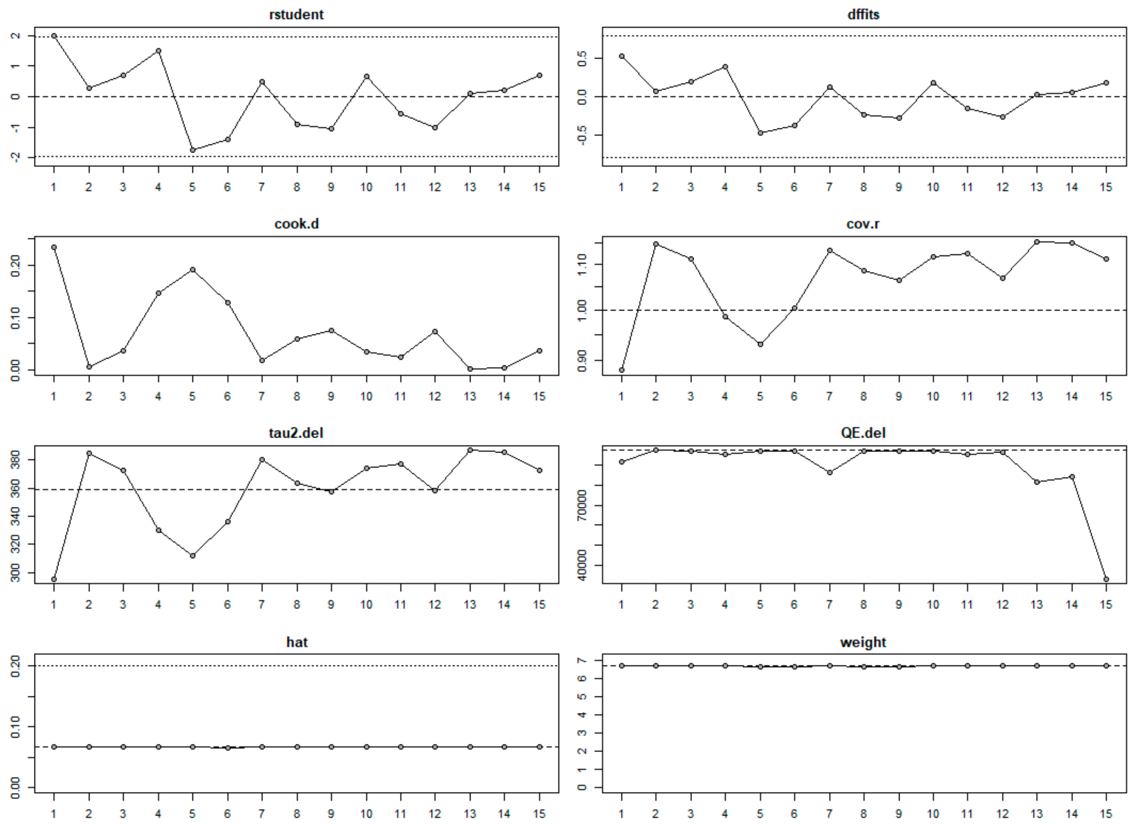


**Figure S2**. Influence analysis of the included studies evaluating the impact of individual studies on the overall meta-analytic results. Metrics such as residual studentized values (rstudent), DFFITS, Cook's distance (Cook.D), variance estimates (tau$^2$), and heterogeneity statistics (QE) were used to assess the influence of each study. The studies are numbered as follows: 1 (Cesarone et al., 2005; pain; Hydroxyethylrutoside), 2 (Cesarone et al., 2006; pain; Pycnogenol), 3 (Cesarone et al., 2006; edema; Pycnogenol), 4 (Cesarone et al., 2006; edema; Pycnogenol), 5 (Belczak et al., 2013; edema; Aminaphthone), 6 (Belczak et al., 2013; edema; Coumarin + Troxerutin), 7 (Cesarone et al., 2005; edema; Hydroxyethylrutoside), 8 (Toledo et al., 2017; edema; Pycnogenol), 9 (Toledo et al., 2017; edema; Pycnogenol), 10 (Cesarone et al., 2006; quality of life; Venoruton), 11 (Belczak et al., 2013; quality of life; Aminaphthone), 12 (Belczak et al., 2013; quality of life; Coumarin + Troxerutin), 13 (Cesarone et al., 2006; resting flux; Pycnogenol), 14 (Cesarone et al., 2006; resting flux; Pycnogenol), and 15 (Cesarone et al., 2005; resting flux; Hydroxyethylrutoside). This analysis highlights studies with significant influence on the pooled effect size and heterogeneity, providing insights into their contribution to the overall meta-analytic conclusions.

**Table S1.** Search Strategies Used for Bibliographic Databases in the Systematic Review.

| DATA BASE | STRATEGY FOR SEARCHING |
|---|---|
| Medline / PubMed | (((((Venous Insufficiency[Title/Abstract]) OR (Insufficiencies, Venous[Title/Abstract])) OR (Insufficiency, Venous[Title/Abstract])) OR (Venous Insufficiencies[Title/Abstract])) AND ((((((Diosmin[Title/Abstract]) OR (Barosmin[Title/Abstract])) OR (Buchu Resin[Title/Abstract])) OR (Resin, Buchu[Title/Abstract])) OR (Venosmine[Title/Abstract])) OR (Daflon[Title/Abstract]))) AND (((((((((Hesperidin[Title/Abstract]) OR (Hesperetin 7-Rhamnoglucoside[Title/Abstract])) OR (7-Rhamnoglucoside, Hesperetin[Title/Abstract])) OR (Hesperetin 7 Rhamnoglucoside[Title/Abstract])) OR (Hesperetin-7-Rutinoside[Title/Abstract])) OR (Hesperetin 7 Rutinoside[Title/Abstract])) OR (Hesperidin 2S[Title/Abstract])) OR (2S, Hesperidin[Title/Abstract])) OR (4H-1- Benzopyran-4-one, 7-((6-O-(6-deoxy-alpha-L-mannopyranosyl)-beta-D-glucopyranosyl)oxy)-2,3-dihydro-5- hydroxy-2-(3-hydroxy-4-methoxyphenyl)-, (S)-[Title/Abstract])). |
| Cochrane library and Embase | "venous insufficiency" in Title Abstract Keyword AND "diosmin" in Title Abstract Keyword AND "hesperidin" in Title Abstract Keyword - with Publication Year from 2000 to 2023, in Trials (Word variations have been searched). |
| Google Scholar | ("Venous Insufficiency" OR "Insufficiencies, Venous" OR "Insufficiency, Venous" OR "Venous Insufficiencies") AND ("Diosmin" OR "Barosmin" OR "Buchu Resin" OR "Resin, Buchu" OR "Venosmine" OR "Daflon") AND ("Hesperidin" OR "Hesperetin 7-Rhamnoglucoside" OR "Hesperetin-7-Rutinoside" OR "Hesperidin 2S" OR "4H-1-Benzopyran-4-one, 7-((6-O-(6-deoxy-alpha-L-mannopyranosyl)-beta-D-glucopyranosyl)oxy)-2,3-dihydro-5-hydroxy-2-(3-hydroxy-4-methoxyphenyl)-, (S)") |
| Scopus | TITLE-ABS("venous insufficiency" OR "insufficiencies, venous" OR "insufficiency, venous" OR "venous insufficiencies") AND TITLE-ABS("diosmin" OR "barosmin" OR "buchu resin" OR "resin, buchu" OR "venosmine" OR "daflon") AND TITLE-ABS("hesperidin" OR "hesperetin 7-rhamnoglucoside" OR "7-rhamnoglucoside, hesperetin" OR "hesperetin 7 rhamnoglucoside" OR "hesperetin-7-rutinoside" OR "hesperidin 2S" OR "4H-1-benzopyran-4-one, 7-((6-O-(6-deoxy-alpha-L-mannopyranosyl)-beta-D-glucopyranosyl)oxy)-2,3-dihydro-5-hydroxy-2-(3-hydroxy-4-methoxyphenyl)-, (S)") |
| Web of Science | ((ALL=("venous insufficiency")) AND ALL=("diosmin")) AND ALL=("hesperidin") |
